# YBX1 Modulates Intimal Hyperplasia by Regulating Expression and Alternative Splicing of Cell Cycle Associated Genes in RASMCs

**DOI:** 10.1111/jcmm.70445

**Published:** 2025-03-05

**Authors:** Yi Huang, Yuheng Wang, Feng Zhu, Chao Guo, Xinyang Zhang, Yiqing Li, Yunfei Chen, Chuanqi Cai, Dan Shang

**Affiliations:** ^1^ Department of Vascular Surgery, Union Hospital, Tongji Medical College Huazhong University of Science and Technology Wuhan China; ^2^ Department of Vascular Surgery Hubei Provincial Hospital of Traditional Chinese Medicine Wuhan China

**Keywords:** alternative splicing, cell cycle, cell migration, intimal hyperplasia, YBX1

## Abstract

YBX1, a DNA‐/RNA‐binding protein, is implicated in various diseases, yet its role in intimal hyperplasia (IH) remains unclear. This study investigates YBX1's function in rat aortic smooth muscle cells (RASMCs) through knockdown experiments. Results show that YBX1 knockdown reduces cell proliferation and migration while inducing apoptosis. ELISA and western blot analyses revealed increased levels of the anti‐inflammatory factor IL10 and markers for phenotypic transformation, Calponin and Myocardin. Transcriptome sequencing identified 1598 differentially expressed genes (DEGs), with 347 upregulated and 1251 downregulated. Upregulated DEGs were linked to pathways like ECM–receptor interaction and Wnt signalling, while downregulated genes involved cell cycle and p53 signalling. Additionally, 629 significant alternative splicing events were noted, primarily affecting pathways related to cell division and migration. Integrated analysis of YBX1‐bound RNAs and RNA‐seq data highlighted key DEGs, such as CCNB1 and TPM1, which are crucial for vascular cell behaviour. This study underscores YBX1's vital role in RASMCs and suggests potential therapeutic targets for IH treatment.

## 1 Introduction

Intimal hyperplasia (IH) is an abnormal cell aggregation phenomenon observed in various developments of vascular remodelling diseases [1]. Vascular remodelling diseases refer to vascular wall thickening and lumen stenosis caused by endothelial damage, proliferation of vascular smooth muscle cells (VSMCs), deposition of extracellular matrix (ECM) and other cardiovascular diseases, including atherosclerosis, venous occlusion and synthetic vascular grafts, in‐stent restenosis and coronary angioplasty [2, 3]. This process is closely related to the increase in cell count. Therefore, its formation is usually related to the activation of vascular cells [4, 5]. There are several types of cells related to the development and continuation of this IH process, mainly VSMCs, adventitia fibroblasts, endothelial progenitor cells and bone marrow–derived progenitor cells, which originate from different intima [6]. VSMCs are crucial for the progression of endometrial sclerosis and the development of neointimal hyperplasia [7]. Finally, the IH process will affect the function of target organs to varying degrees. There are many studies on the mechanism of vascular IH, such as endothelial cell injury [8], migration and proliferation of VSMCs [9], lipid deposition formation [10], inflammatory response and formation of autoimmune complexes [6, 11]. However, due to the complexity of its pathogenesis, it has not been thoroughly elucidated to this day.

RNA‐binding proteins (RBPs) play very important roles in the process of co‐transcription and post‐transcriptional regulation, mainly by combining with RNA, affecting the production, modification, splicing, stability, transportation, intracellular localization, translation and degradation of mRNA. RBPs play important regulatory roles in almost all physiological and pathological situations [12, 13]. Among them, the YBX1 protein, also known as YB‐1, has both DNA and RNA functions and is involved in many cellular processes, including regulation of transcription and translation, regulation of pre‐mRNA splicing, DNA repair and mRNA packaging and stability [14, 15]. Recent studies demonstrated that YBX1 is a 5‐methylcytosine (m^5^C) reader that recognises m^5^C sites and recruits other proteins to stabilise mRNA levels [16, 17]. YBX1 can also regulate the transcription process as a DNA‐binding protein [18, 19, 20]. The function of YBX1 in VSMCs has been preliminarily studied [21, 22, 23], but there is still limited research on how it functions as an RBP. Therefore, it is necessary to conduct in‐depth research on the regulatory targets of YBX1, which can help deepen the understanding of its functional mechanism in vascular intimal hyperplasia.

Based on previous research, we speculate that YBX1 plays an important role in vascular intimal hyperplasia and that it may affect the occurrence and development of vascular intimal hyperplasia in rat aortic smooth muscle cells (RASMCs) by affecting the expression of target genes and alternative splicing. In this project, we knocked down YBX1 by small hairpin RNA (shRNA) in RASMCs and obtained transcriptome sequencing data (RNA‐seq) affected by YBX1. The potential target genes for expression levels and alternative splicing patterns regulated by YBX1 in RASMC and their functions were analysed. Meanwhile, we also explored the RNA immunoprecipitation and sequencing (RIP‐seq) data of YBX1 using the published dataset GSE159153 [24]. By integrating the RNA‐seq and RIP‐seq datasets, we deeply explored the underlying mechanisms of how YBX1 regulates gene expression and alternative splicing. In summary, we highlight the important functions of YBX1 in IH and identified the potential downstream targets of YBX1, which can be used as candidate therapeutic targets for IH and other cardiovascular diseases in the future.

## 2 Materials and Methods

### 2.1 shRNA Information

All lentivirus shRNA duplexes were purchased from Genepharma (Suzhou, China), non‐targeting control shRNA (shNC): 5′‐TTCTCCGAACGTGTCACGT‐3′ (sense) and shRNA targeting Ybx1 (shYbx1): 5′‐ATGCGCGTCGACCACAGTATT‐3′ (sense). The lentiviral vector used in this study was LV‐3 (pGLVH1/GFP + Puro).

### 2.2 Cell Culture and Transfections

RASMC cells (CP‐R076, Procell, Wuhan, China) were cultured at 37°C with 5% CO_2_ in an appropriate complete growth medium (CM‐R076, Procell Life Science & Technology Co. Ltd., China) with 10% foetal bovine serum (FBS) (10091148, Gibco, China), 100 μg/mL streptomycin and 100 U/mL penicillin (SV30010, Hyclone, USA). RASMCs were infected by the shYbx1 and shNC lentivirus duplexes with MOI = 300. Stable cell lines were obtained by screening with 2 μg/mL puromycin, and cells were harvested for RT‐qPCR and western blot analyses.

### 2.3 Assessment of Gene Expression by RT‐qPCR

The cDNA synthesis was done by reverse transcription kit (R323‐01, Vazyme, China) at 42°C for 5 min，37°C for 15 min and 85°C for 5 s performed on the thermocycler (T100, Bio‐Rad, USA). qPCR was performed on the ABI QuantStudio 5, followed by denaturing at 95°C for 10 min, 40 cycles of denaturing at 95°C for 15 s and annealing and extension at 60°C for 1 min. Each sample had three technical replicates. The concentration of each transcript was then normalised to GAPDH (glyceraldehyde‐3‐phosphate dehydrogenase) and mRNA level using the 2^−ΔΔCT^ method for analysis [25]. Comparisons were performed with the paired Student's *t*‐test by using GraphPad Prism software (Version 8.0, San Diego, CA). Primers for PCR experiments are presented in Table S1.

### 2.4 Western Blot

RASMC cells were lysed in ice‐cold RIPA buffer (PR20001, Proteintech, China) supplemented with a protease inhibitor cocktail (4693116001, Sigma, USA) and incubated on ice for 30 min. Samples were boiled for 10 min in boiling water with protein loading buffer (P1040, Solarbio, China), loaded onto a 10% SDS‐PAGE gel and transferred onto 0.45 mm PVDF membranes (ISEQ00010, Millipore, USA). The PVDF membranes were then blocked for 1 h at room temperature and incubated overnight at 4°C with primary antibodies against YBX1 (anti‐YBX1, 1:1000, antibody produced in rabbit, ab239875, Abcam, UK) and GAPDH (1:5000, antibody produced in mouse, 60004‐1‐lg, Proteintech, China), followed by an incubation with horseradish peroxidase‐conjugated secondary antibody (anti‐rabbit, 1:10,000, SA00001‐2, Proteintech, China, or anti‐mouse, 1:10,000, AS003, ABclonal, China) for 45 min at room temperature. Then, membranes were visualised using the enhanced ECL reagent (P0018FM, Beyotime, China) through chemiluminescence.

### 2.5 Cell Proliferation Assay

The cell proliferation assay was conducted using a Cell Counting kit‐8 (CCK‐8, HY‐K0301, MCE, Shanghai, China). Briefly, RASMC cells were seeded at 35,000 cells/well in 24‐well culture plates. Cells treated with an equal volume of phosphate‐buffered saline (PBS) served as controls and vials without cells were used as blank controls. Following cultured for 24 h, 10 μL CCK‐8 solution was added to the culture medium and incubated for an additional 3 h. Subsequent to this, the optical density of the cells was measured with a microplate reader (ELX800, BioTek, USA) at an absorbance of 450 nm. The cell proliferation rate was calculated using the following formula: proliferation rate = (experimental OD value − blank OD value)/(control OD value − blank OD value) × 100%.

### 2.6 Annexin V Apoptosis Assay

To detect tumour cell apoptosis, an Annexin V‐Alexa Fluor647/PI apoptosis detection kit (40304ES60, YEASEN, China) was used according to the manufacturer's instructions. Specifically, MDA‐MB‐231 cells were seeded into six‐well plates, cultured for 48 h and transfected with siRNA of SF3B6. Cells treated with an equal volume of PBS served as a control. The treated and control cells were mixed with 5 μL Annexin V‐Alexa Fluor647 and 10 μL of 20 μg/mL PI reagents. The cells were then incubated at room temperature in the dark for 10–15 min. Then the samples were subjected to flow cytometry (FACSCanto, BD, USA) analysis to detect cell apoptosis levels.

### 2.7 Cell Migration Assay

In vitro migration assays were performed using transwell chambers (3422, Corning, USA). A total of 5 × 10^4^ RASMC cells in 0.2 mL serum‐free medium were added to the transwell chambers with an 8 μm filter and then the chambers were inserted in a medium with 600 uL 10% FBS (10091148, Gibco, China) serving as a chemoattractant in the lower chamber and incubated for 24 h at 37°C and 5% CO_2_. Cells remaining on the upper membrane surface of the inserts were then removed with a cotton swab, and the total number of cells that migrated into the lower chamber was fixed by 4% paraformaldehyde (P0099, Beyotime, China) for 30 min and then stained with 0.1% crystal violet (C0121, Beyotime, China). The migrating cells were observed and counted using an inverted microscope (MF52‐N, Mshot, China) at 200× magnification.

### 2.8 ELISA Assays of IL‐10

Following incubation with the ligands, the concentrations of rat IL‐10 in the culture medium were determined by the use of ELISA in accordance with the manufacturer's instructions.

### 2.9 RNA Extraction and Sequencing

RNA‐seq assays were performed by Wuhan Ruixing Biotechnology Co. Ltd. For each sample, 1 μg of total RNA was treated with RQ1 DNase (M6101, Promega, USA) to remove DNA before being used for directional RNA‐seq library preparation by VAHTS Universal V8 RNA‐seq Library Prep Kit for Illumina (NR605, Vazyme, China). The mRNAs were captured by VAHTS mRNA Capture Beads (N401, Vazyme, China). Fragmented RNAs were converted into double‐strand cDNA. Following end repair and A tailing, the DNAs were ligated to VAHTS RNA Multiplex Oligos Set 1 for Illumina (N323, Vazyme, China). The ligated products were amplified, purified, quantified and stored at −80°C before sequencing. The strand marked with dUTP (the second cDNA strand) was not amplified, allowing strand‐specific sequencing.

For high‐throughput sequencing, the libraries were prepared following the manufacturer's instructions and applied to Illumina NovaSeq 6000 system for 150 nt paired‐end sequencing.

### 2.10 RNA‐Seq Raw Data Clean and Alignment

Raw reads containing more than 2‐N bases were first discarded. Then, adaptors and low‐quality bases were trimmed from raw sequencing reads using FASTX‐Toolkit (Version 0.0.13). The short reads less than 16 nt were also dropped. After that, clean reads were aligned to the mRatBN7.2 genome by HISAT2 [26], allowing four mismatches. Uniquely mapped reads were used for counting number of gene reads and FPKM calculation (fragments per kilobase of transcript per million fragments mapped) [27].

### 2.11 Differentially Expressed Genes (DEGs) Analysis

The R bioconductor package DESeq2 [28] was utilised to screen out the DEGs. The *p*‐value < 0.05 and fold change > 1.5 or < 0.67 were set as the cut‐off criteria for identifying DEGs.

### 2.12 Alternative Splicing Analysis

The alternative splicing events (ASEs) and regulated alternative splicing events (RASEs) between the samples were defined and quantified by using the ABLas pipeline as described previously [29]. In brief, ABLas detection of 10 types of ASEs was based on the splice junction reads, including exon skipping (ES), alternative 5′ splice site (A5SS), alternative 3′ splice site (A3SS), mutually exclusive exons (MXE), mutually exclusive 5′ UTRs (5pMXE), mutually exclusive 3′ UTRs (3pMXE), cassette exon, A3SS&ES and A5SS&ES.

To assess RBP‐regulated ASE, the Student's *t*‐test was performed to evaluate the significance of the ratio alteration of AS events. Those events that were significant at P‐value cut‐off corresponding to a false discovery rate cut‐off of 5% were considered as RBP‐regulated ASEs.

### 2.13 RIP‐Seq Data Analysis

The accession number of the Gene Expression Omnibus (GEO) database was GSE159153. Public sequencing data were obtained from the Sequence Read Archive (SRA). SRA Run data files were transformed into fastq format with the NCBI SRA Tool fastq‐dump. Low‐quality bases were discarded using the FASTX‐Toolkit (v.0.0.13). Then, the clean reads were analysed with FastQC.

After reads were aligned onto the genome with HISAT2 [26], the unique comparison on the genome was finally obtained, and the comparison result of PCR duplicates was removed. Then, two software programs, Piranha and ABLIRC, were used to perform peak calling. Piranha has been described elsewhere [30]. ‘ABLIRC’ strategy was used to identify the binding regions of GRCh38 on the genome as previously described [29]. The process of peak calling was as follows: first, the whole genome was scanned with a window of 5 bp and a step of 5 bp from the beginning of each chromosome. A peak was identified by requiring that the depth of the first window was 2.5 times for eight consecutive windows on the genome or the median depth was greater than 50. When the depth of eight consecutive windows was less than 4% of the maximum depth of this peak, the peak ended. At the same time, reads on each gene were randomly distributed to each gene for 500 times, and the frequency of peak depth of each peak was counted, so as to conduct significance analysis on the identified peak and screen out the peak with significant peak (*p*‐value < 0.05) or peak with maximum depth of a certain degree (≥ 10). Then, with the input samples as the control, the abundance difference analysis was conducted for the locations of these peaks, and the peak with IP abundance greater than four times (adjustable parameter) of input abundance was screened as the final combination peak. The target genes of IP were finally determined by the peaks, and the binding motifs of the IP protein were called by HOMER software [31].

### 2.14 RIP‐qPCR Experiment

RASMCs were lysed in ice‐cold wash buffer (1 × PBS, 0.5% SDS, 0.5% NP‐40 and 0.5% sodium deoxycholate) supplemented with a 400 U/mL RNase inhibitor (2313A, Takara, Japan) and protease inhibitor cocktail (B14001, Bimake, China) and incubated on ice for 30 min. We added RQ I (M6101, Promega, USA) to a final concentration of 0.1 U/μL and incubated in a heat block for 30 min at 37°C. The mixture was then vibrated vigorously and centrifuged at 13,000× *g* at 4°C for 15 min to remove cell debris.

For immunoprecipitation, the supernatant was incubated overnight at 4°C with 5 μg YBX1 antibody (Y0396‐200UL, Sigma, USA) and control IgG antibody (AC005, ABclonal, China). The immunoprecipitates were further incubated with protein A/G Dynabeads (26162, Thermo Scientific, USA) for 2 h at 4°C. After applying to the magnet and removing the supernatants, the beads were washed six times with NT buffer (50 mM Tris 7.4, 150 mM NaCl, 1 mM Mgcl_2_, 0.05% NP‐40). The beads were resuspended in elution buffer (50 mM Tris 8.0, 10 mM EDTA and 1% SDS). The suspension was incubated for 30 min in a heat block at 70°C to release the immunoprecipitated RBP with RNA and vortex. We removed the magnetic beads on the separator, transferred the supernatant to a clean 1.5 mL microfuge tube, added Proteinase K (B600169‐0002, Sangon Biotech, China) into the 10% input (without immunoprecipitatation) and immunoprecipitated YBX1 with RNA, with a final concentration of 1.2 mg/mL. The suspension was incubated for 120 min at 55°C. The RNA was purified with phenol:chloroform:isopentyl alcohol (25:24:1, pH < 5) reagent (p1011, Solarbio, China). RIP‐qPCR was performed following the RT‐qPCR experiment.

### 2.15 Functional Enrichment Analysis

To sort out functional categories of DEGs, Gene Ontology (GO) terms and KEGG pathways were identified using the KOBAS 2.0 server [32]. Hypergeometric test and Benjamini–Hochberg FDR controlling procedure were used to define the enrichment of each term.

## 3 Results

YBX1 knockdown inhibits cell proliferation, apoptosis and cell metastasis of RASMC cells.

**FIGURE 1:**
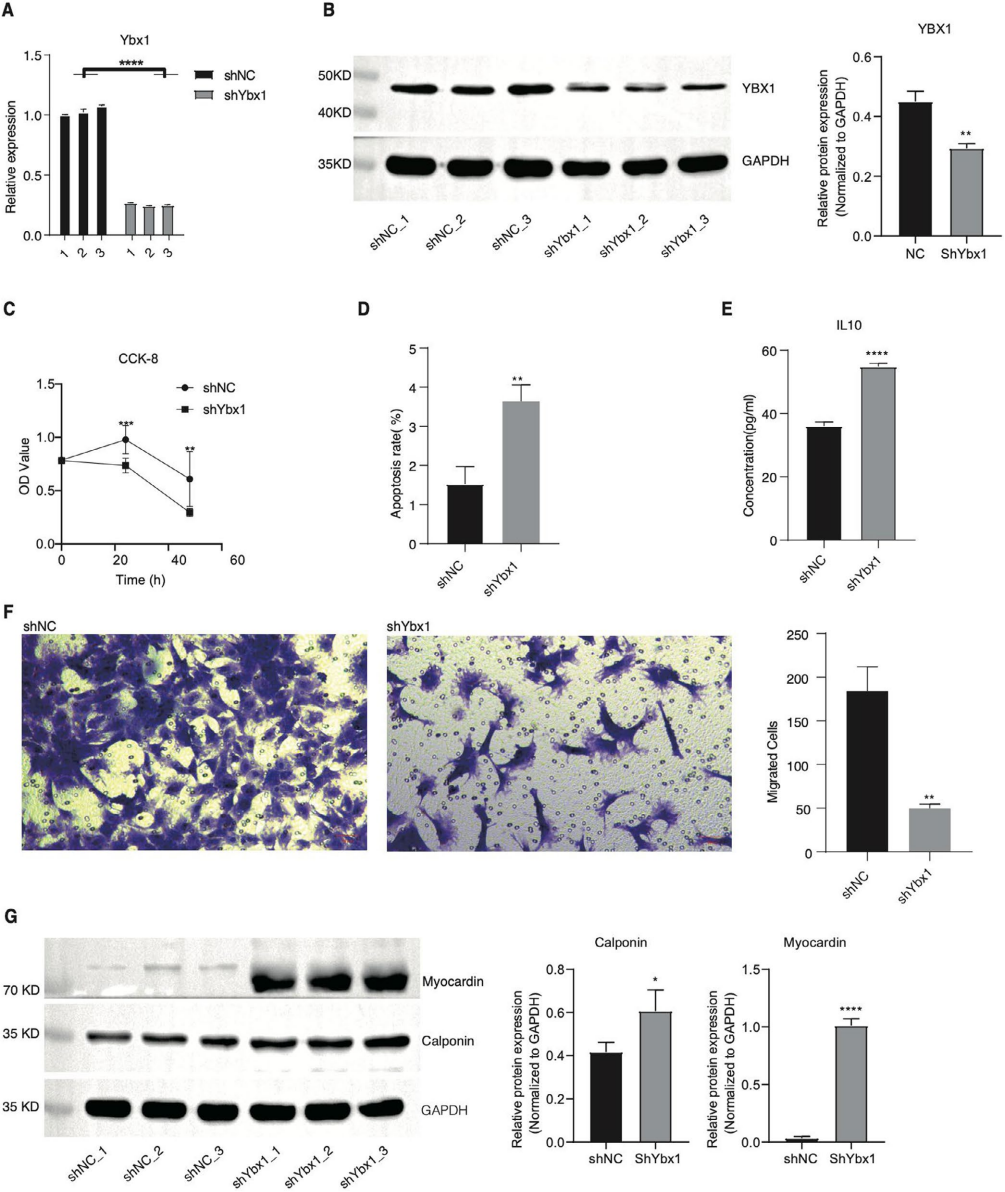
Ybx1 knockdown inhibits cell proliferation and metastasis, and promotes apoptosis of RASMC cells. (A) RT‐qPCR showing expression of Ybx1 mRNA in Ybx1 knockdown RASMC cells. (B) YBX1 protein detection by western blot in Ybx1 knockdown RASMC cells. (C) RASMC cells were transfected with either vector control or shRNA‐Ybx1 for 24 h, and cell viability was determined by the CCK‐8 assay. (D) Effect of Ybx1 knockdown on apoptosis regulation in RASMC cells. Apoptosis was detected by an Annexin V‐Fluor647/PI flow cytometric apoptosis assay. (E) An ELISA assay was performed to examine the expression levels of IL10 in vector control and Ybx1 knockdown RASMC cells. (F) The migration capacity of vector control and Ybx1 knockdown RASMC cells was tested with transwell assays. Scale bar: 50 μm. (G) Western blot showing the increased expression levels of myocardin and calponin in shYbx1 samples. The right panel was the quantitative result of greyscale values. Student's *t*‐test, *N* = 3; *p < 0.05; **p < 0.01; ***p < 0.001; ****p < 0.0001.

As YBX1 plays important roles in gene expression and multiple biological processes [14], we decided to explore how YBX1 modulates the phenotype and gene expression pattern of RASMCs. We knocked down Ybx1 expression in RASMCs using short hairpin RNA (shRNA). After transfecting shRNA into RASMCs, we found the RNA level of Ybx1 was significantly repressed in shYbx1 samples compared with that in NC samples (Figure 1A). This inhibition of Ybx1 was also confirmed by the WB experiment with an obvious difference between shYbx1 and shNC samples (Figure 1B), indicating the successful knockdown of Ybx1. We then assessed the cellular phenotype difference between these two groups. The cellular viability of RASMCs was significantly decreased in shYbx1 samples, while the apoptotic level was significantly increased in shYbx1 samples (Figure 1C,D), suggesting that Ybx1 knockdown repressed the growth and viability of RASMCs, which was consistent with previous results in cancer cells [33]. Meanwhile, we detected that IL10, which can repress proinflammatory responses and maintain the integrity and homeostasis of tissue epithelial layers [34], was increased in shYbx1 samples by the ELISA test (Figure 1E). Further experiments demonstrated that shYbx1 significantly repressed the migration capacity of RASMCs (Figure 1F). Finally, we analysed the expression pattern of phenotypic transformation markers of VSMCs, including calponin (CNN1) and myocardin (MYOCD), which participate in the differentiation and phenotypic switching of smooth muscle cells [35, 36]. We found these two markers were significantly increased in shYbx1 samples (Figure 1G), indicating that the differentiation of RASMCs was enhanced by shYbx1. In summary, these results showed that Ybx1 significantly modulates the phenotypes of RASMCs, which is probably associated with the IH phenomenon in vascular diseases.

### 3.1 YBX1 Regulates Expression of Genes Associated With IH in RASMC Cells

**FIGURE 2:**
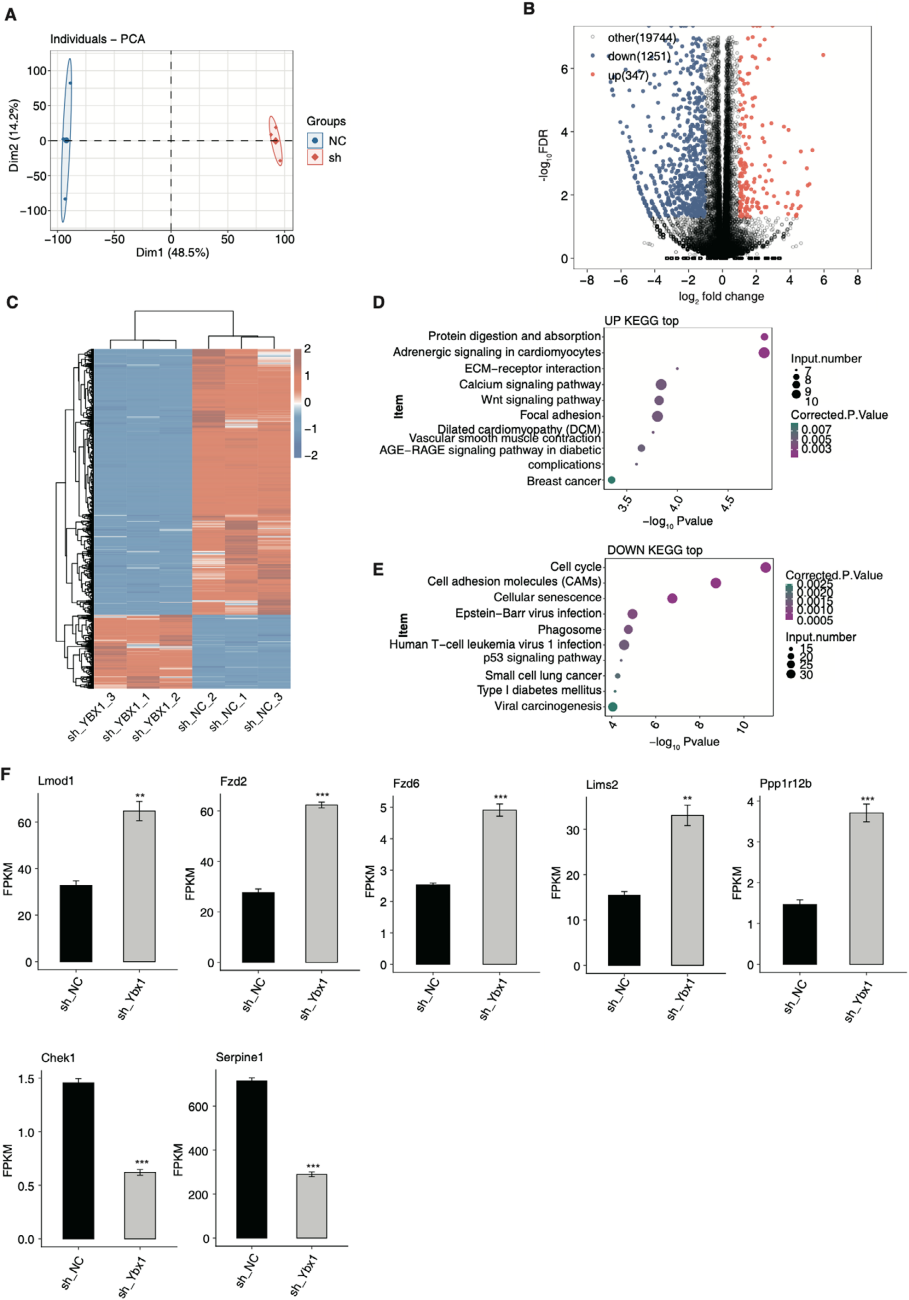
YBX1 regulates gene expression in RASMC cells in intima hyperplasia. (A) Principal component analysis (PCA) of samples after normalising expression levels of all genes. The ellipse for each group is the confidence ellipse. (B) Volcano plot showing all differentially expressed genes (DEGs) between shYbx1 and NC samples. Red indicates genes with upregulated expression. Blue indicates genes with downregulated expression. (C) Hierarchical clustering heat map showing expression levels of all DEGs. DEGs were clustered on the basis of K‐means. (D) Bubble plot exhibiting the most enriched KEGG analysis of upregulated genes. (E) Bubble plot exhibiting the most enriched KEGG analysis of downregulated genes. (F) Bar plot showing the expression pattern and statistical difference of DEGs from RNA sequencing. Error bars represent mean ± SEM. *N* = 3; **p‐value < 0.01, ***p‐value < 0.001.

To further illustrate how YBX1 modulates the phenotypes of RASMCs, we performed global transcriptome sequencing analysis (RNA‐seq) for the identification of downstream targets of YBX1. By calculating all the expressed genes, principal component analysis (PCA) illustrated a clear separation between shYbx1 and shNC samples at the first component, which can explain 48.5% of total variation (Figure 2A), indicating a global transcriptome difference induced by shYbx1. Then we performed DEG analysis and identified the DEG set with two‐fold change and 0.05 FDR as thresholds. It is very interesting that shYbx1 resulted in more downregulated DEGs (1251) than upregulated DEGs (347), suggesting that shYbx1 repressed the expression of a large part of genes (Figure 2B). Meanwhile, the upregulated and downregulated DEGs also showed a consistent expression pattern among the three replicates of both shYbx1 and shNC (Figure 2C). By performing KEGG enrichment analysis for upregulated and downregulated DEGs, we found that upregulated DEGs were enriched in protein digestion and absorption, adrenergic signalling in cardiomyocytes, ECM–receptor interaction, Wnt signalling pathway and focal adhesion pathways (Figure 2D). For downregulated DEGs, cell cycle, cell adhesion molecules (CAMs), cellular senescence, Epstein–Barr virus infection and phagosome were the top enriched pathways (Figure 2E). The enriched GO BP pathways for upregulated and downregulated DEGs also demonstrated similar pathways in the KEGG analysis (Figure S1). The enriched cell cycle pathway for downregulated DEGs was consistent with the decreased proliferation level of RASMCs by shYbx1 (Figure 1C). At the same time, the enriched ECM‐associated pathways, including ECM–receptor interaction and CAMs, were also the molecular causes of IH [37]. Finally, we selected several DEGs that were highly associated with the pathogenesis or development of vascular diseases to illustrate their dysregulated expression levels between shYbx1 and shNC samples, including Lmod1, Fzd2, Fzd6, Lims2, Ppp1r12b, Chek1 and Serpine1 (Figure 2F). In summary, these results suggest the profound transcriptional regulation of YBX1 in RASMCs and its potential function in IH formation.

### 3.2 YBX1 Regulates Alternative Splicing of Genes Associated With IH in RASMCs

**FIGURE 3:**
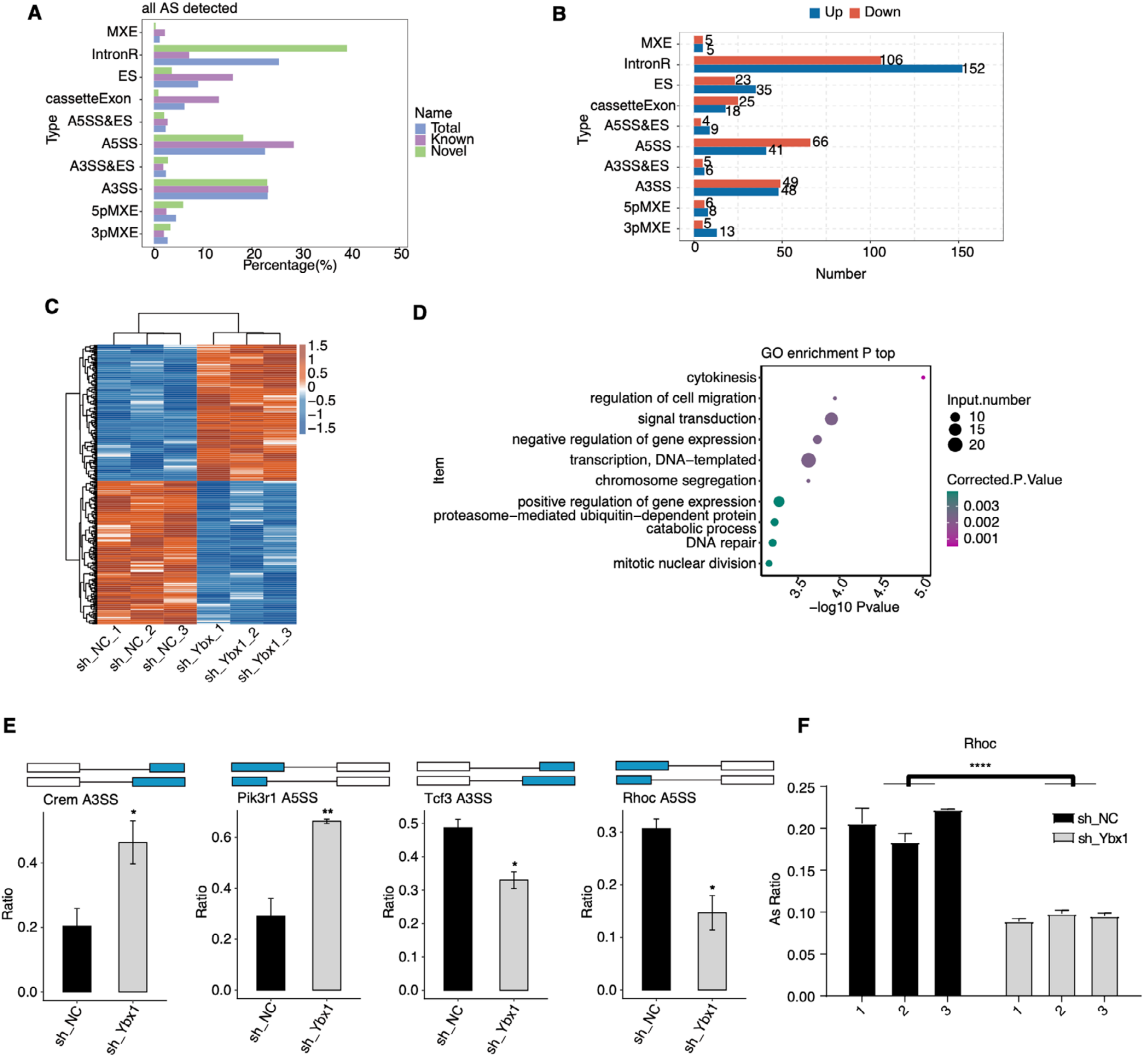
YBX1 regulates gene alternative splicing in RASMC cells in intima hyperplasia. (A) The bar plot shows the number of all alternative splicing events (ASEs). *X*‐axis: all ASE number. *Y*‐axis: the different types of AS events. (B) The bar plot shows the number of all significant regulated alternative splicing events (RASEs) between shYbx1 and NC samples. *X*‐axis: all RASE numbers. *Y*‐axis: the different types of AS events. (C) Hierarchical clustering heat map showing expression levels of all RASEs. DEGs were clustered on the basis of K‐means. (D) Bubble plot exhibiting the most enriched GO biological process results of the regulated alternative splicing genes (RASGs). (E)The schematic diagrams depict the structures of RASEs (top). RNA‐seq validation of the splicing ratio profile of the splicing event from Crem, Pik3r1, Tcf3 and Rhoc. Error bars represent mean ± SEM. *N* = 3; ***p‐value < 0.001, **p‐value < 0.01, *p‐value < 0.05. (F) The bar plot showing the RT‐qPCR results for A5SS RASE of *Rhoc*. Error bars represent mean ± SEM. *N* = 3; ****p‐value < 0.0001.

Previous studies also reported the primary RNA splicing influence of YBX1 in multiple conditions [15, 21]. Thus, we investigated the alternative splicing pattern and change between shYbx1 and shNC samples. We analysed splicing changes following *Ybx1* knockdown and identified the novel, known and total AS events. A5SS, A3SS, IntronR, ES and cassetteExon were the top five AS types (Figure 3A). The most significant AS event was intron retention among the 629 significantly regulated AS events (RASEs) between shYbx1 and shNC samples (Figure 3B). The clear separation between shYbx1 and shNC was found by plotting and clustering the AS ratio of these RASEs (Figure 3C). To analyse the functions of these RASE genes (RASGs), GO biological process analysis revealed that RASGs were significantly enriched in cytokinesis, mitotic nuclear division, regulation of cell migration and other pathways (Figure 3D), indicating that these RASGs may also contribute to the altered cell proliferation and migration of RASMCs by shYbx1. We finally presented four RASEs/RASGs that were associated with the proliferation and migration of cells, including *Crem*, *Pik3r1*, *Tcf3* and *Rhoc* (Figure 3E). Then we performed RT‐qPCR to validate these RASEs. The RT‐qPCR results showed that the ratio of the Rhoc A5SS event was significantly decreased in shYbx1 samples, consistent with changes in RNA‐seq (Figure 3F). In summary, these results demonstrated that the YBX1‐modulated AS profile can also influence the phenotypes of RASMCs and participate in the development of IH.

### 3.3 YBX1 Binds and Regulates mRNA Expression in RASMC Cells

**FIGURE 4:**
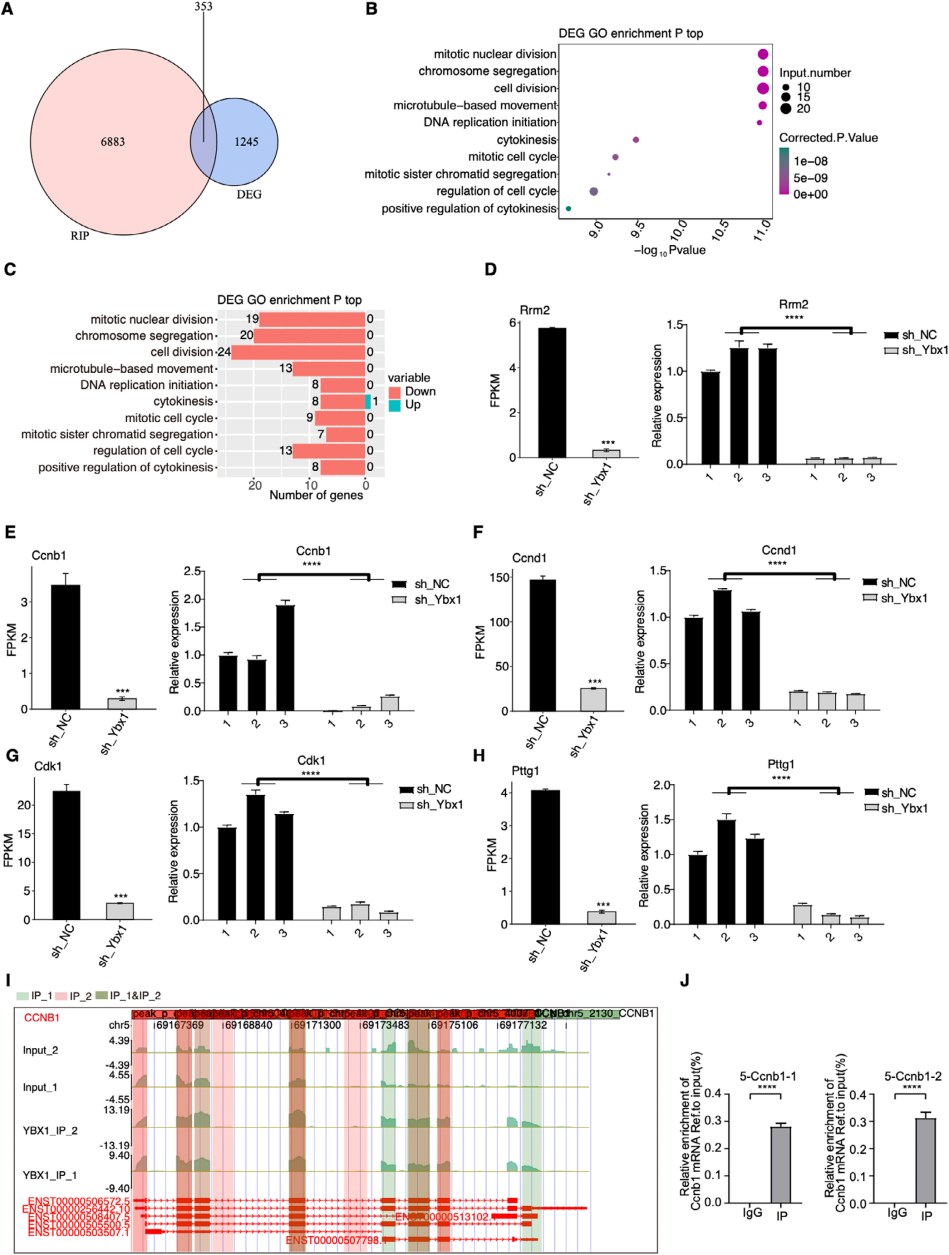
YBX1 binds and regulates mRNA expression in RASMC cells. (A)Venn diagram showing the overlap of DEGs and RIP‐seq. (B) Bubble plot exhibiting the most enriched GO biological process results of the overlapping DEGs. (C) The bar plot showing the number of up‐or downregulated genes in (B). (D–H) Bar plot showing the expression pattern and statistical difference of DEGs for six important genes. Error bars represent mean ± SEM. *N* = 3; ***p‐value < 0.001. (I) YBX1 binding peak genes of CCNB1. IGV‐Sashimi plot showing the peak reads and binding sites across mRNA; the green and red panels represent the positions of peaks. Reads distribution of the bound gene is plotted in the upper panel and the transcripts of each gene are shown below. (J) Bar plot showing the relative binding enrichment of YBX1 on Ccnb1 transcript. Error bars represent mean ± SEM. *N* = 3; ****p‐value < 0.0001.

To further investigate the potential regulatory role of YBX1 as an RBP, we downloaded and re‐analysed a set of RIP‐seq data (GSE159153) for YBX1‐Flag in HEK293T cells [24]. The abundance profile of identified genes was distinct between YBX1‐IP and input samples (Figure S2A). After identifying the bound peaks by YBX1, we found that the peaks were mainly from CDS and intron regions (Figure S2B), and showed significant overlap between the two replicates (Figure S2C, *p*‐value = 0, hypergeometric test). Functional enrichment analysis of YBX1‐bound RNA genes showed that they were mainly enriched in cell cycle and RNA processing–associated pathways (Figure S2D,E). Then we integrated YBX1‐bound genes with the DEGs identified from our RNA‐seq data and found 353 overlapped genes (Figure 4A), suggesting that YBX1 interacts with the RNAs of these genes to affect their expression or even function in RASMCs. Meanwhile, we found that most of these overlapped DEGs were downregulated in shYbx1 samples (Figure 4B). We performed GO functional enrichment analysis on these 353 genes and showed that these genes were mainly enriched in pathways related to cell cycle and proliferation, such as cell division, mitotic nuclear division and chromosome segregation (Figure 4B). By plotting the DEG number in each pathway, we found that all the DEGs were downregulated by shYbx1 except one in cytokinesis (Figure 4C). Next, we focused on the top genes associated with disease. According to previous reports, the functions of *CCNB1*, *CCND1*, *RRM2*, *CDK1* and *PTTG1* may be related to cell proliferation and migration [38, 39], and all expressions of them were significantly downregulated following *YBX1* knockdown and were validated by RT‐qPCR (Figure 4D–H), which implies YBX1 plays an important role in IH. The distribution maps of reads showed that YBX1 binds to the RNA of *CCNB1* and *CCND1* (Figures 4I and S2F), which were further validated by RIP‐qPCR in RASMCs (Figures 4J and S2G). Above all, the results demonstrated that YBX1 can regulate gene expression by binding to their transcripts, which were associated with the pathogenesis of IH.

### 3.4 YBX1 Binds to and Regulates mRNA Alternative Splicing in RASMCs

**FIGURE 5:**
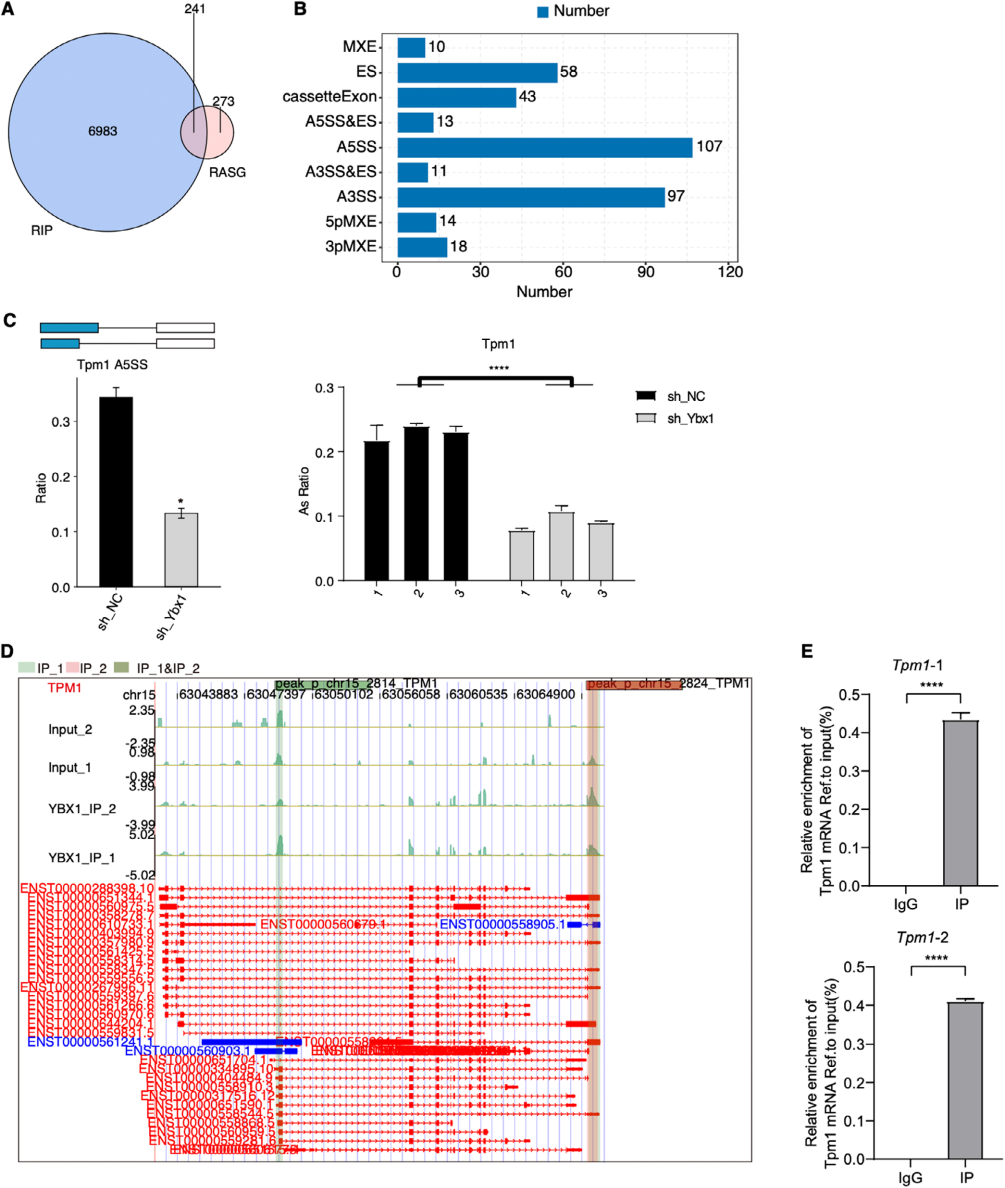
YBX1 binds and regulates mRNA alternative splicing in RASMCs. (A) Venn diagram showing the overlapped genes between YBX1‐bound genes and RASGs. (B) The bars show the distributed number of overlapped RASEs. (C) Bar plot showing the expression pattern and statistical difference of Tpm1 A5SS by RNA‐seq (left panel) and RT‐qPCR (right panel) experiments. Error bars represent mean ± SEM. *N* = 3; *p‐value < 0.05; ****p‐value < 0.0001. (D) YBX1 binds to the mRNAs of TPM1. IGV‐Sashimi plot showing binding sites across mRNA. The peak ranges are highlighted with pink or green colours. (E) Bar plot showing the relative binding enrichment of YBX1 on Tpm1 transcript. Error bars represent mean ± SEM. *N* = 3; ****p‐value < 0.0001.

Additionally, we want to decipher how YBX1 influences the AS pattern of its bound transcripts. Then we combined peak genes bound by YBX1 from RIP‐seq and RASGs from RNA‐seq, and found 241 overlapped RASGs that were YBX1 regulated (Figure 5A, *p*‐value = 1.96e−10, hypergeometric test), indicating that YBX1 can modulate the AS pattern of genes by binding to their transcripts. By analysing the types of overlapped RASEs, we found A5SS and A3SS were the most prevalent types according to their classification (Figure 5B). Among the YBX1‐bound RASEs, we found that the A5SS event of *Tpm1* was significantly suppressed by shYbx1, and we validated this AS event by RT‐qPCR (Figure 5C). TPM1, whose expression was modulated by microRNA‐21, can significantly regulate the proliferation and migration of VSMCs [40]. To have a clear visualisation of the bound density of YBX1 on the *TPM1* transcript, we plotted the mapped reads density of RIP‐seq on *TIM1* transcript and found two obviously enriched binding sites of YBX1 on *TPM1* (Figure 5D). To confirm the binding ability of YBX1 on the TPM1 transcript, we performed a RIP‐qPCR experiment in RASMCs and validated the significant YBX1–*TPM1* interaction for the two binding sites (Figure 5E). In summary, our results highlight the AS regulatory function of YBX1 by binding to RNA transcripts, which is tightly associated with IH development.

## 4 Discussion

Many reports have shown that alternative splicing and dysregulation of the cell cycle promote the occurrence and progression of multiple diseases. However, there are few studies on RBPs related to IH. Recent studies proved that vascular injury activates the RBP ILF3 of VSMC, which promotes intima formation [41]. LncPSR and arteridin regulate the gene expression and phenotypic transition of VSMCs by forming complexes with YBX1 [42]. In this study, we demonstrated that YBX1 can act as both a transcriptional regulator and a splicing factor to modulate the cellular phenotypes of RASMCs, probably participating in the pathogenesis of intimal hyperplasia. At the same time, by integrating the RNA binding profile of YBX1, we predicted the underlying mechanism by which YBX1 affects gene expression and alternative splicing. In summary, these results highlight the important roles of YBX1 in RASMCs and its influence on IH formation and development.

After the knockdown of *Ybx1* in RASMCs, we found that shYbx1 inhibited cell proliferation and migration, promoted apoptosis and increased the expression of the anti‐inflammatory factor IL10. One recent study demonstrated that YBX1 was highly expressed in human endothelial cells and was increased in murine plaques compared to wild‐type vessels [43]. IL10 inhibits smooth muscle cell activation and reduces IH [44, 45], consistent with our experimental results. YBX1 knockdown caused expression changes in a large part of the genes. Upregulated genes were mainly enriched in ECM–receptor interaction, Wnt signalling pathway, focal adhesion, vascular smooth muscle contraction and other pathways, while downregulated genes were primarily enriched in cell cycle, CAM, p53 signalling pathway and other pathways. As a DNA‐binding protein and RBP, we showed that shYBX1 affected the expression of many genes. Among them, *LMOD1* may affect the phenotypic transformation of smooth muscle cells [46]. *FZD2* and *FZD6* play an important role in Wnt signalling, regulating epithelial–mesenchymal transformation (EMT) and tumour progression [47, 48]. LIMS2, PPP1R12B, CHEK1 and SERPINE1 are all associated with cell migration and invasion [49, 50]. Based on the above cognition, YBX1 may have an important function in IH by modulating related biological processes through interacting with other molecules [42]. In summary, YBX1 may affect the cell proliferation and migration of RASMCs by regulating the expression of these genes, thereby affecting the progression of IH.

There are lots of differential ASEs regulated by shYBX1, yielding the RNA binding ability of YBX1. The GO functions of these RASGs are enriched in cytokinesis; transcription; DNA‐templated, mitotic nuclear division and other pathways, most of which were related to cell cycle. CREM, RHOC, PIK3R1 and TCF3 are involved in the regulation of proliferation and or apoptosis of VSMCs [51, 52, 53]. Finally, we integrated the RIP‐seq data of YBX1 to identify its binding targets. Although the RIP‐seq and RNA‐seq datasets did not come from the same cell line and can bring discrepancy for the findings, we detected hundreds of genes that were bound by YBX1 and showed significant difference at expression or alternative splicing levels, indicating that YBX1 has profound influence on its bound targets, thus modulating the pathogenesis or development of IH. Meanwhile, we performed RIP‐qPCR experiment on RASMCs and validated YBX1 binding ability on several transcripts, confirming the potential regulation of YBX1 on its bound transcripts.

The overlapped DEGs and RASGs of RIP‐seq and RNA‐seq were mainly enriched in cell cycle–related pathways. The DEGs *CCNB1*, *CCND1*, *PLK1*, *RRM2*, *CDK1* and *PTTG1* and the RASG *TPM1* both affect the proliferation of cells and are associated with IH [54, 55, 56]. CCND1 may also participate in the proliferation and migration of VSMCs in vitro and intimal hyperplasia in vivo [57]. Cyclin‐dependent protein kinases (CDKs), including CDK1, have been extensively discussed in the proliferation and migration of VSMCs, as well as their inhibitors [58]. PTTG1 is a regulator of VSMC migration and phenotype through MAPK signalling [59]. TPM1, whose AS pattern was modulated by YBX1, participates in the proliferation and migration of VSMCs [60]. Another study also demonstrated that the DHX9 and YBX1 complex can modulate the AS pattern of KLF5 mRNA, thus altering the phenotypic transformation of VSMCs and involving aortic dissection disease [21], suggesting a possible regulatory manner for YBX1 in AS by interacting with other proteins. These results together indicate that YBX1 influences the proliferation and apoptosis of RASMCs by regulating the expression and alternative splicing of cell cycle–related genes through interactions with other key factors, yielding the essential functions and therapeutic potential of YBX1 in IH.

Although we have preliminarily confirmed the role of YBX1 in IH, more complete and rational experimental studies on the mechanism of its function are lacking. For instance, we have not conducted animal model and comprehensive molecular experiment verification, and we have not thoroughly investigated how YBX1 regulates downstream targets to affect the occurrence and even the progress of intimal hyperplasia. The two sets of sequencing data we analysed were not from the same cell or even the same species, which may have made our conclusions less accurate.

In conclusion, we suggest that YBX1 may influence the progression of intimal hyperplasia by regulating the expression and alternative splicing of genes related to cell migration and the cell cycle. Furthermore, our study provides a reliable basis for the investigation of the function of YBX1 in intimal hyperplasia.

## Author Contributions

**Yi Huang:** conceptualization (equal), methodology (equal), resources (equal), writing – original draft (equal), writing – review and editing (equal). **Yuheng Wang:** conceptualization (equal), data curation (equal), formal analysis (equal), methodology (equal), resources (equal), writing – original draft (equal), writing – review and editing (equal). **Feng Zhu:** conceptualization (equal), data curation (equal), formal analysis (equal), methodology (equal), resources (equal), writing – original draft (equal), writing – review and editing (equal). **Chao Guo:** conceptualization (equal), data curation (equal), formal analysis (equal), methodology (equal), resources (equal), writing – original draft (equal), writing – review and editing (equal). **Xinyang Zhang:** conceptualization (equal), methodology (equal), resources (equal), writing – original draft (equal), writing – review and editing (equal). **Yiqing Li:** conceptualization (equal), data curation (equal), formal analysis (equal), investigation (equal), methodology (equal), project administration (equal), resources (equal), software (equal), supervision (equal), validation (equal), visualization (equal). **Yunfei Chen:** conceptualization (equal), data curation (equal), formal analysis (equal), investigation (equal), methodology (equal), resources (equal), software (equal), supervision (equal). **Chuanqi Cai:** conceptualization (equal), data curation (equal), formal analysis (equal), investigation (equal), methodology (equal), project administration (equal), resources (equal), software (equal), supervision (equal), validation (equal), visualization (equal). **Dan Shang:** conceptualization (equal), funding acquisition (equal), investigation (equal), methodology (equal), project administration (equal), resources (equal), supervision (equal), validation (equal).

## Consent

The retrospective study was approved by the institutional research committee of our institution with a waiver of informed consent.

## Conflicts of Interest

The authors declare no conflicts of interest.

## Supporting information


Figure S1.

Table S1.

## Data Availability

All data generated or analysed during this study are included in this published article.

## References

[1] P.‐K. Tran , K. Tran‐Lundmark , R. Soininen , K. Tryggvason , J. Thyberg , and U. Hedin , “Increased Intimal Hyperplasia and Smooth Muscle Cell Proliferation in Transgenic Mice With Heparan Sulfate—Deficient Perlecan,” Circulation Research 94, no. 4 (2004): 550–558. 14739157 10.1161/01.RES.0000117772.86853.34

[2] X. Wang and R. A. Khalil , “Matrix Metalloproteinases, Vascular Remodeling, and Vascular Disease,” Advances in Pharmacology 81 (2018): 241–330, 10.1016/bs.apha.2017.08.002. 29310800 PMC5765875

[3] T. Melnik , O. Jordan , J.‐M. Corpataux , F. Delie , and F. Saucy , “Pharmacological Prevention of Intimal Hyperplasia: A State‐Of‐The‐Art Review,” Pharmacology & Therapeutics 235 (2022): 108157. 35183591 10.1016/j.pharmthera.2022.108157

[4] S. F. Louis , P. J. E. Zahradka , and C. Cardiology , “Vascular Smooth Muscle Cell Motility: From Migration to Invasion,” Experimental & Clinical Cardiology 15, no. 4 (2010): e75–e85. 21264073 PMC3016065

[5] J. C. Jennette and J. R. Stone , “Diseases of Medium‐Sized and Small Vessels,” in Cellular and Molecular Pathobiology of Cardiovascular Disease (Elsevier, 2014), 197–219.

[6] R. Che Man , N. Sulaiman , M. F. Ishak , R. Bt Hj Idrus , M. R. Abdul Rahman , and M. D. J. I. Yazid , “The Effects of Pro‐Inflammatory and Anti‐Inflammatory Agents for the Suppression of Intimal Hyperplasia: An Evidence‐Based Review,” International Journal of Environmental Research and Public Health 17, no. 21 (2020): 7825, 10.3390/ijerph17217825. 33114632 PMC7672569

[7] K. Xu , M. K. Al‐Ani , X. Pan , Q. Chi , N. Dong , and X. Qiu , “Plant‐Derived Products for Treatment of Vascular Intima Hyperplasia Selectively Inhibit Vascular Smooth Muscle Cell Functions,” Evidence‐Based Complementary and Alternative Medicine 2018 (2018): 3549312. 30405738 10.1155/2018/3549312 PMC6201497

[8] H. Sun , R. Morihara , T. Feng , et al., “Human Cord Blood‐Endothelial Progenitor Cells Alleviate Intimal Hyperplasia of Arterial Damage in a Rat Stroke Model,” Cell Transplantation 32 (2023): 9636897231193069, 10.1177/09636897231193069. 37615293 PMC10467372

[9] S. Deglise , C. Bechelli , and F. Allagnat , “Vascular Smooth Muscle Cells in Intimal Hyperplasia, an Update,” Frontiers in Physiology 13 (2022): 1081881, 10.3389/fphys.2022.1081881. 36685215 PMC9845604

[10] Y. X. Liu , P. Z. Yuan , J. H. Wu , and B. Hu , “Lipid Accumulation and Novel Insight Into Vascular Smooth Muscle Cells in Atherosclerosis,” Journal of Molecular Medicine (Berlin, Germany) 99, no. 11 (2021): 1511–1526, 10.1007/s00109-021-02109-8. 34345929

[11] L. Bogdanov , D. Shishkova , R. Mukhamadiyarov , et al., “Excessive Adventitial and Perivascular Vascularisation Correlates With Vascular Inflammation and Intimal Hyperplasia,” International Journal of Molecular Sciences 23, no. 20 (2022), 10.3390/ijms232012156. PMC9603343 36293013

[12] R. Xiao , J. Chen , Z. Liang , et al., “Pervasive Chromatin‐RNA Binding Protein Interactions Enable RNA‐Based Regulation of Transcription,” Cell 178, no. 1 (2019): 107–121.e18. 31251911 10.1016/j.cell.2019.06.001 PMC6760001

[13] J. I. Perez‐Perri , M. Noerenberg , W. Kamel , et al., “Global Analysis of RNA‐Binding Protein Dynamics by Comparative and Enhanced RNA Interactome Capture,” Nature Protocols 16, no. 1 (2021): 27–60. 33208978 10.1038/s41596-020-00404-1 PMC7116560

[14] D. N. Lyabin , I. A. Eliseeva , and L. P. Ovchinnikov , “YB‐1 Protein: Functions and Regulation,” Wiley Interdisciplinary Reviews‐RNA Impact Factor 5, no. 1 (2014): 95–110, 10.1002/wrna.1200. 24217978

[15] E. Stickeler , S. D. Fraser , A. Honig , A. L. Chen , S. M. Berget , and T. A. Cooper , “The RNA Binding Protein YB‐1 Binds A/C‐Rich Exon Enhancers and Stimulates Splicing of the CD44 Alternative Exon v4,” EMBO Journal 20, no. 14 (2001): 3821–3830, 10.1093/emboj/20.14.3821. 11447123 PMC125550

[16] X. Chen , A. Li , B. F. Sun , et al., “5‐Methylcytosine Promotes Pathogenesis of Bladder Cancer Through Stabilizing mRNAs,” Nature Cell Biology 21, no. 8 (2019): 978–990, 10.1038/s41556-019-0361-y. 31358969

[17] Y. Yang , L. Wang , X. Han , et al., “RNA 5‐Methylcytosine Facilitates the Maternal‐To‐Zygotic Transition by Preventing Maternal mRNA Decay,” Molecular Cell 75, no. 6 (2019): 1188–1202 e11, 10.1016/j.molcel.2019.06.033. 31399345

[18] R. Chattopadhyay , S. Das , A. K. Maiti , et al., “Regulatory Role of Human AP‐Endonuclease (APE1/Ref‐1) in YB‐1‐Mediated Activation of the Multidrug Resistance Gene MDR1,” Molecular and Cellular Biology 28, no. 23 (2008): 7066–7080, 10.1128/MCB.00244-08. 18809583 PMC2593380

[19] J. T. Norman , G. E. Lindahl , K. Shakib , A. En‐Nia , E. Yilmaz , and P. R. Mertens , “The Y‐Box Binding Protein YB‐1 Suppresses Collagen Alpha 1(I) Gene Transcription via an Evolutionarily Conserved Regulatory Element in the Proximal Promoter,” Journal of Biological Chemistry 276, no. 32 (2001): 29880–29890, 10.1074/jbc.M103145200. 11395503

[20] T. M. Campbell , M. A. A. Castro , K. G. de Oliveira , B. A. J. Ponder , and K. B. Meyer , “ERalpha Binding by Transcription Factors NFIB and YBX1 Enables FGFR2 Signaling to Modulate Estrogen Responsiveness in Breast Cancer,” Cancer Research 78, no. 2 (2018): 410–421, 10.1158/0008-5472.CAN-17-1153. 29180470 PMC5774586

[21] W. Huan , J. Zhang , Y. Li , and K. Zhi , “Involvement of DHX9/YB‐1 Complex Induced Alternative Splicing of Kruppel‐Like Factor 5 mRNA in Phenotypic Transformation of Vascular Smooth Muscle Cells,” American Journal of Physiology. Cell Physiology 317, no. 2 (2019): C262–C269, 10.1152/ajpcell.00067.2019. 31116584

[22] R. Krohn , U. Raffetseder , I. Bot , et al., “Y‐Box Binding Protein‐1 Controls CC Chemokine Ligand‐5 (CCL5) Expression in Smooth Muscle Cells and Contributes to Neointima Formation in Atherosclerosis‐Prone Mice,” Circulation 116, no. 16 (2007): 1812–1820, 10.1161/CIRCULATIONAHA.107.708016. 17893273

[23] L. Dhawan , B. Liu , A. Pytlak , S. Kulshrestha , B. C. Blaxall , and M. B. Taubman , “Y‐Box Binding Protein 1 and RNase UK114 Mediate Monocyte Chemoattractant Protein 1 mRNA Stability in Vascular Smooth Muscle Cells,” Molecular and Cellular Biology 32, no. 18 (2012): 3768–3775, 10.1128/MCB.00846-12. 22801372 PMC3430190

[24] M. Feng , X. Xie , G. Han , et al., “YBX1 Is Required for Maintaining Myeloid Leukemia Cell Survival by Regulating BCL2 Stability in an m6A‐Dependent Manner,” Blood, the Journal of the American Society of Hematology 138, no. 1 (2021): 71–85. 10.1182/blood.2020009676 PMC8667054 33763698

[25] K. J. Livak and T. D. Schmittgen , “Analysis of Relative Gene Expression Data Using Real‐Time Quantitative PCR and the 2(‐Delta Delta C(T)) Method,” Methods 25, no. 4 (2001): 402–408, 10.1006/meth.2001.1262. 11846609

[26] D. Kim , B. Langmead , and S. L. Salzberg , “HISAT: A Fast Spliced Aligner With Low Memory Requirements,” Nature Methods 12, no. 4 (2015): 357–360. 25751142 10.1038/nmeth.3317 PMC4655817

[27] C. Trapnell , B. A. Williams , G. Pertea , et al., “Transcript Assembly and Quantification by RNA‐Seq Reveals Unannotated Transcripts and Isoform Switching During Cell Differentiation,” Nature Biotechnology 28, no. 5 (2010): 511–515, 10.1038/nbt.1621. PMC3146043 20436464

[28] M. I. Love , W. Huber , and S. Anders , “Moderated Estimation of Fold Change and Dispersion for RNA‐Seq Data With DESeq2,” Genome Biology 15, no. 12 (2014): 1–21. 10.1186/s13059-014-0550-8 PMC4302049 25516281

[29] H. Xia , D. Chen , Q. Wu , et al., “CELF1 Preferentially Binds to Exon‐Intron Boundary and Regulates Alternative Splicing in HeLa Cells,” Biochimica et Biophysica Acta (BBA)‐Gene Regulatory Mechanisms 1860, no. 9 (2017): 911–921. 28733224 10.1016/j.bbagrm.2017.07.004

[30] P. J. Uren , E. Bahrami‐Samani , S. C. Burns , et al., “Site Identification in High‐Throughput RNA–Protein Interaction Data,” Bioinformatics 28, no. 23 (2012): 3013–3020. 23024010 10.1093/bioinformatics/bts569 PMC3509493

[31] S. Heinz , C. Benner , N. Spann , et al., “Simple Combinations of Lineage‐Determining Transcription Factors Prime Cis‐Regulatory Elements Required for Macrophage and B Cell Identities,” Molecular Cell 38, no. 4 (2010): 576–589. 20513432 10.1016/j.molcel.2010.05.004 PMC2898526

[32] C. Xie , X. Mao , J. Huang , et al., “KOBAS 2.0: A Web Server for Annotation and Identification of Enriched Pathways and Diseases,” Nucleic Acids Research 39, no. suppl_2 (2011): W316–W322, 10.1093/nar/gkr483. 21715386 PMC3125809

[33] Y. Ban , Y. Tan , X. Li , et al., “RNA‐Binding Protein YBX1 Promotes Cell Proliferation and Invasiveness of Nasopharyngeal Carcinoma Cells via Binding to AURKA mRNA,” Journal of Cancer 12, no. 11 (2021): 3315–3324, 10.7150/jca.56262. 33976741 PMC8100805

[34] W. Ouyang , S. Rutz , N. K. Crellin , P. A. Valdez , and S. G. Hymowitz , “Regulation and Functions of the IL‐10 Family of Cytokines in Inflammation and Disease,” Annual Review of Immunology 29 (2011): 71–109, 10.1146/annurev-immunol-031210-101312. 21166540

[35] M. Gimona , M. Herzog , J. Vandekerckhove , and J. V. Small , “Smooth Muscle Specific Expression of Calponin,” FEBS Letters 274, no. 1–2 (1990): 159–162, 10.1016/0014-5793(90)81353-p. 2253769

[36] M. Ackers‐Johnson , A. Talasila , A. P. Sage , et al., “Myocardin Regulates Vascular Smooth Muscle Cell Inflammatory Activation and Disease,” Arteriosclerosis, Thrombosis, and Vascular Biology 35, no. 4 (2015): 817–828, 10.1161/ATVBAHA.114.305218. 25614278 PMC4390125

[37] A. C. Newby and A. B. Zaltsman , “Molecular Mechanisms in Intimal Hyperplasia,” Journal of Pathology 190, no. 3 (2000): 300–309, 10.1002/(sici)1096-9896(200002)190:3<300::Aid-path596>3.0.Co;2-i. 10685064

[38] M. Fischer , A. E. Schade , T. B. Branigan , G. A. Muller , and J. A. DeCaprio , “Coordinating Gene Expression During the Cell Cycle,” Trends in Biochemical Sciences 47, no. 12 (2022): 1009–1022, 10.1016/j.tibs.2022.06.007. 35835684

[39] M. L. Whitfield , G. Sherlock , A. J. Saldanha , et al., “Identification of Genes Periodically Expressed in the Human Cell Cycle and Their Expression in Tumors,” Molecular Biology of the Cell 13, no. 6 (2002): 1977–2000. 12058064 10.1091/mbc.02-02-0030. PMC117619

[40] M. Wang , W. Li , G. Q. Chang , et al., “MicroRNA‐21 Regulates Vascular Smooth Muscle Cell Function via Targeting Tropomyosin 1 in Arteriosclerosis Obliterans of Lower Extremities,” Arteriosclerosis, Thrombosis, and Vascular Biology 31, no. 9 (2011): 2044–2053, 10.1161/ATVBAHA.111.229559. 21817107

[41] Y. M. Hou , B. H. Xu , Q. T. Zhang , et al., “Deficiency of Smooth Muscle Cell ILF3 Alleviates Intimal Hyperplasia via HMGB1 mRNA Degradation‐Mediated Regulation of the STAT3/DUSP16 Axis,” Journal of Molecular and Cellular Cardiology 190 (2024): 62–75, 10.1016/j.yjmcc.2024.04.004. 38583797

[42] J. Yu , W. Wang , J. Yang , et al., “LncRNA PSR Regulates Vascular Remodeling Through Encoding a Novel Protein Arteridin,” Circulation Research 131, no. 9 (2022): 768–787, 10.1161/CIRCRESAHA.122.321080. 36134578 PMC9588624

[43] V. Fürle , U. Laufs , J.‐N. Boeckel , and V. Filipova , “YBX Genes Regulate Endothelial‐To‐Mesenchymal Transition (EndMT),” Atherosclerosis 379 (2023): S22.

[44] L. J. Feldman , L. Aguirre , M. Ziol , et al., “Interleukin‐10 Inhibits Intimal Hyperplasia After Angioplasty or Stent Implantation in Hypercholesterolemic Rabbits,” Circulation 101, no. 8 (2000): 908–916, 10.1161/01.cir.101.8.908. 10694531

[45] M. Mazighi , A. Pellé , W. Gonzalez , et al., “IL‐10 Inhibits Vascular Smooth Muscle Cell Activation In Vitro and In Vivo,” American Journal of Physiology. Heart and Circulatory Physiology 287, no. 2 (2004): H866–H871, 10.1152/ajpheart.00918.2003. 15072956

[46] L. Perisic Matic , U. Rykaczewska , A. Razuvaev , et al., “Phenotypic Modulation of Smooth Muscle Cells in Atherosclerosis Is Associated With Downregulation of LMOD1, SYNPO2, PDLIM7, PLN, and SYNM,” Arteriosclerosis, Thrombosis, and Vascular Biology 36, no. 9 (2016): 1947–1961, 10.1161/atvbaha.116.307893. 27470516 PMC8991816

[47] B. Dong , L. Simonson , S. Vold , et al., “FZD6 Promotes Melanoma Cell Invasion but Not Proliferation by Regulating Canonical Wnt Signaling and Epithelial–Mesenchymal Transition,” Journal of Investigative Dermatology 143, no. 4 (2023): 621–629.e6, 10.1016/j.jid.2022.09.658. 36368445 PMC10292634

[48] L. Huang , E. L. Luo , J. Xie , et al., “FZD2 Regulates Cell Proliferation and Invasion in Tongue Squamous Cell Carcinoma,” International Journal of Biological Sciences 15, no. 11 (2019): 2330–2339, 10.7150/ijbs.33881. 31595151 PMC6775310

[49] C. Xin , Z. Chao , W. Xian , W. Zhonggao , and L. Tao , “The Phosphorylation of CHK1 at Ser345 Regulates the Phenotypic Switching of Vascular Smooth Muscle Cells Both In Vitro and In Vivo,” Atherosclerosis 313 (2020): 50–59. 33027721 10.1016/j.atherosclerosis.2020.09.014

[50] X. Zhang , Y. Zhou , Y. Ye , et al., “Human Umbilical Cord Mesenchymal Stem Cell‐Derived Exosomal microRNA‐148a‐3p Inhibits Neointimal Hyperplasia by Targeting Serpine1,” Archives of Biochemistry and Biophysics 719 (2022): 109155, 10.1016/j.abb.2022.109155. 35218720

[51] C. Hudson , T. E. Kimura , A. Duggirala , G. B. Sala‐Newby , A. C. Newby , and M. Bond , “Dual Role of CREB in the Regulation of VSMC Proliferation: Mode of Activation Determines Pro‐Or Anti‐Mitogenic Function,” Scientific Reports 8, no. 1 (2018): 4904. 29559698 10.1038/s41598-018-23199-4 PMC5861041

[52] T. M. Seasholtz , M. Majumdar , D. D. Kaplan , and J. H. Brown , “Rho and Rho Kinase Mediate Thrombin‐Stimulated Vascular Smooth Muscle Cell DNA Synthesis and Migration,” Circulation Research 84, no. 10 (1999): 1186–1193, 10.1161/01.res.84.10.1186. 10347093

[53] S. Bi , Q. Peng , W. Liu , C. Zhang , and Z. Liu , “MicroRNA‐342‐5p Activates the Akt Signaling Pathway by Downregulating PIK3R1 to Modify the Proliferation and Differentiation of Vascular Smooth Muscle Cells,” Experimental and Therapeutic Medicine 20, no. 6 (2020): 239, 10.3892/etm.2020.9369. 33193844 PMC7646700

[54] C. Silvestre‐Roig , P. Fernandez , M. L. Mansego , et al., “Genetic Variants in CCNB1 Associated With Differential Gene Transcription and Risk of Coronary In‐Stent Restenosis,” Circulation. Cardiovascular Genetics 7, no. 1 (2014): 59–70, 10.1161/CIRCGENETICS.113.000305. 24395923

[55] Y. Wu , J. Sun , A. Li , and D. Chen , “The Promoted Delivery of RRM2 siRNA to Vascular Smooth Muscle Cells Through Liposome‐Polycation‐DNA Complex Conjugated With Cell Penetrating Peptides,” Biomedicine & Pharmacotherapy 103 (2018): 982–988, 10.1016/j.biopha.2018.03.068. 29710515

[56] J. L. Wilson , L. Wang , Z. Zhang , N. S. Hill , and P. Polgar , “Participation of PLK1 and FOXM1 in the Hyperplastic Proliferation of Pulmonary Artery Smooth Muscle Cells in Pulmonary Arterial Hypertension,” PLoS One 14, no. 8 (2019): e0221728, 10.1371/journal.pone.0221728. 31437238 PMC6705859

[57] S. Fan , C. Wang , K. Huang , and M. Liang , “Myricanol Inhibits Platelet Derived Growth Factor‐BB‐Induced Vascular Smooth Muscle Cells Proliferation and Migration In Vitro and Intimal Hyperplasia In Vivo by Targeting the Platelet‐Derived Growth Factor Receptor‐Beta and NF‐kappaB Signaling,” Frontiers in Physiology 12 (2021): 790345, 10.3389/fphys.2021.790345. 35185599 PMC8850918

[58] V. Andres , “Control of Vascular Cell Proliferation and Migration by Cyclin‐Dependent Kinase Signalling: New Perspectives and Therapeutic Potential,” Cardiovascular Research 63, no. 1 (2004): 11–21, 10.1016/j.cardiores.2004.02.009. 15194457

[59] C. Hu , W. Huang , N. Xiong , and X. Liu , “SP1‐Mediated Transcriptional Activation of PTTG1 Regulates the Migration and Phenotypic Switching of Aortic Vascular Smooth Muscle Cells in Aortic Dissection Through MAPK Signaling,” Archives of Biochemistry and Biophysics 711 (2021): 109007, 10.1016/j.abb.2021.109007. 34400144

[60] S. Jia , W. D. Ma , C. Y. Zhang , et al., “Tanshinone IIA Attenuates High Glucose Induced Human VSMC Proliferation and Migration Through miR‐21‐5p‐Mediated Tropomyosin 1 Downregulation,” Archives of Biochemistry and Biophysics 677 (2019): 108154, 10.1016/j.abb.2019.108154. 31672498

