# Supplementary material for: YBX1 Modulates Intimal Hyperplasia by Regulating Expression and Alternative Splicing of Cell Cycle Associated Genes in RASMCs
Source: J Cell Mol Med. 2025 Mar 5;29(5):e70445. doi: 10.1111/jcmm.70445 (PMC11882473; doi:10.1111/jcmm.70445)
Supplement: Supplementary file 1 — Figure S1. [file JCMM-29-e70445-s001.docx]

**Supplementary Figures**


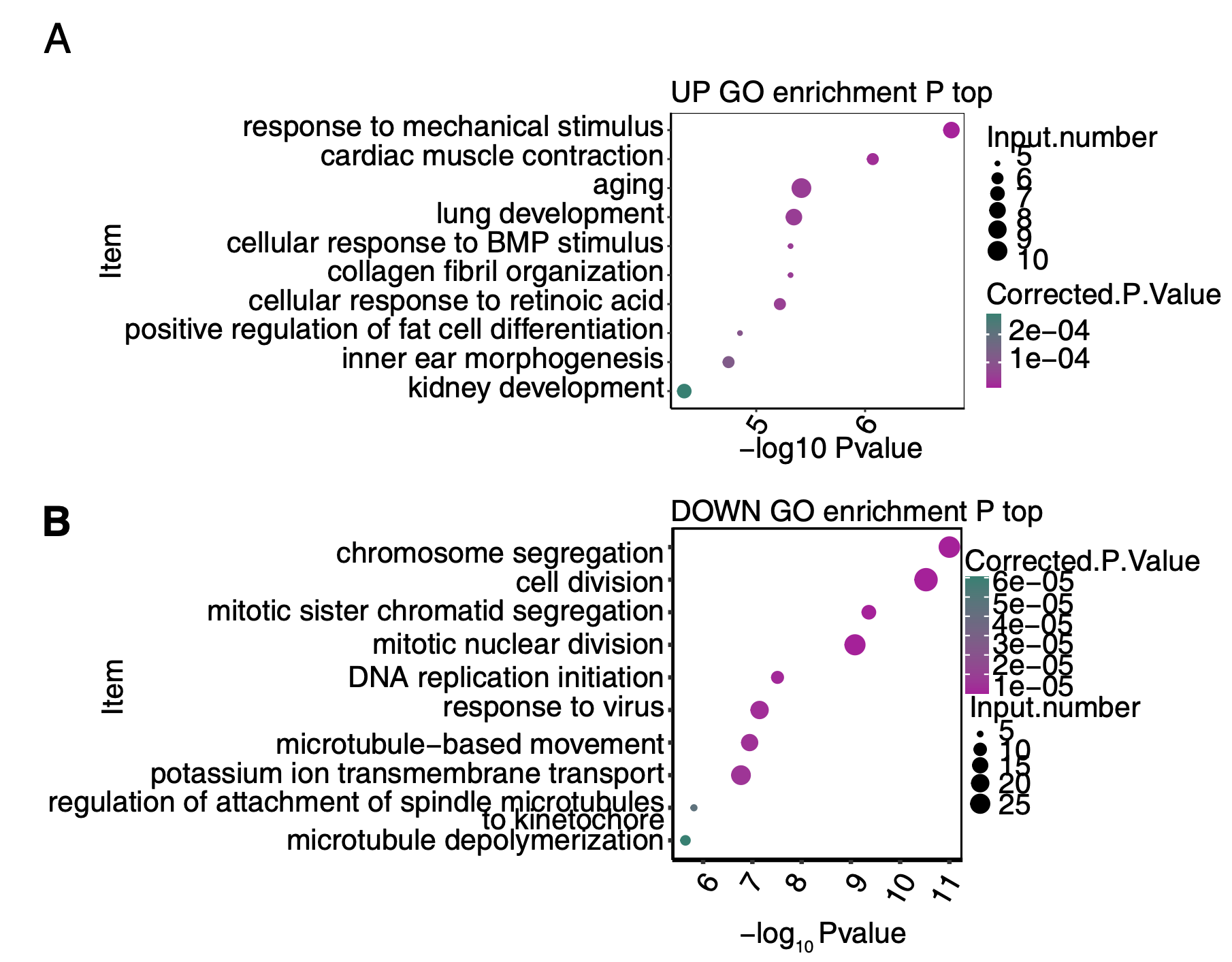


**Figure S1. The functional pathway analysis for DEGs regulated by shYbx1 in RASMCs.** (**A**) Bubble plot exhibiting the most enriched GO biological process results of the up-regulated DEGs. (**B**) Bubble plot exhibiting the most enriched GO biological process results of the down-regulated DEGs.


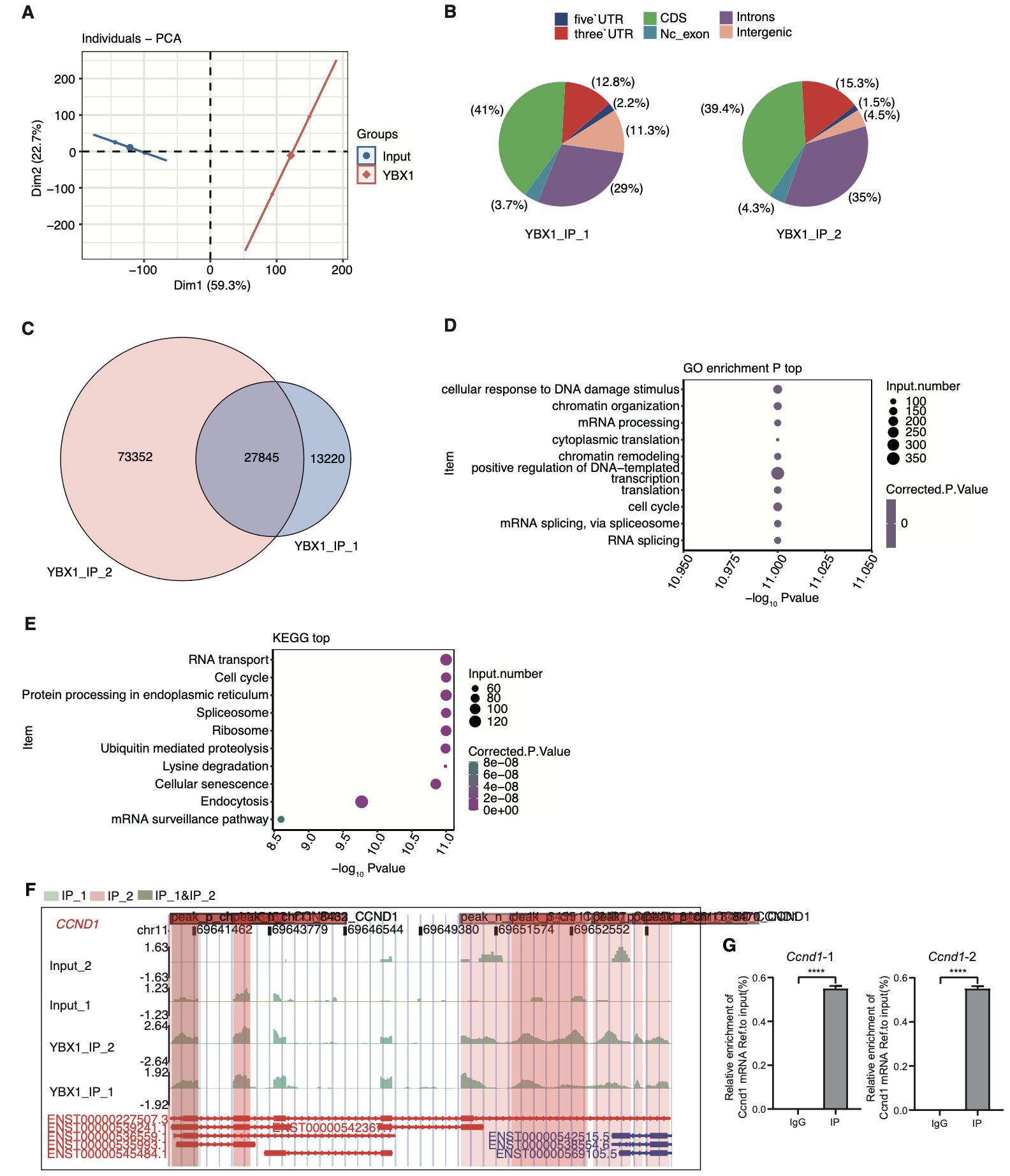


**Figure S2. The global profile and functional analysis for genes bound by YBX1. (A)** Principal component analysis (PCA) of samples after normalizing all genes expression levels. The ellipse for each group is the confidence ellipse. **(B)** Peak distribution across the reference genome. **(C)** The Venn diagram showing the overlap of repeated binding peaks obtained by positional information between the two RNA immunoprecipitation sequencing (RIP-seq) sample replicates. **(D)** Bubble plot exhibiting the most enriched GO biological process results of genes where the overlapping peaks are located. **(E)** The top 10 representative KEGG pathways of genes where the overlapping peaks are located. (**F**) YBX1 binding peak genes of *CCND1*. IGV-sashimi plot showing the peaks reads and binding sites across mRNA, the green and red panels represent the position of peaks. Reads distribution of bound gene is plotted in the up panel and the transcripts of each gene are shown below. (**G**) Bar plot showing the relative binding enrichment of YBX1 on *Ccnd1* transcript. Error bars represent mean ± SEM. N = 3; **** *P*-value < 0.0001.
