# Supplementary material for: YBX1 Modulates Intimal Hyperplasia by Regulating Expression and Alternative Splicing of Cell Cycle Associated Genes in RASMCs
Source: J Cell Mol Med. 2025 Mar 5;29(5):e70445. doi: 10.1111/jcmm.70445 (PMC11882473; doi:10.1111/jcmm.70445)
Supplement: Supplementary file 2 — Table S1. [file JCMM-29-e70445-s002.docx]

**Table S1. The primer sequences for RT-qPCR and RIP-qPCR experiments.**

| RT-qPCR primers for DEGs | |
| --- | --- |
| Ccnd1-F: | TGGACACAGCAGCCCTCAA |
| Ccnd1-R: | TAATGTAAAGCCAGCCGCAATG |
| Ccnb1-F: | CAGACGAGGTAGTCCATTC |
| Ccnb1-R: | ACGATCAAGAACAAGTATGC |
| Rrm2-F: | AACTATGTAACTCTGGCTTGGC |
| Rrm2-R: | TGGTCACAGAAACCTTAATCCC |
| Pttg1-F: | CACACTCAGAGTCGGCTGTT |
| Pttg1-R: | GGTTTCAACGCCACGAGTC |
| Cdk1-F: | ATGATCCAGCCAAACGAATCTC |
| Cdk1-R: | AGACAGGAAGAGCCAACAGTAA |
| Ybx1-F | GAGAGGATGGCAATGAAGA |
| Ybx1-R | TGCGTCGGTAATTGAAGT |
| RT-qPCR primers for AS events | |
| Rhoc-M-F: | GGAGCAGAAGTTTCAGTCAT |
| Rhoc-AS-F: | CGGGCGGGGATTTCAGTCAT |
| Rhoc-M/AS-R: | ACGATGAGGAGGCAGGTCTT |
| Tpm1-M/AS-F: | ATCCAACTCCTCCTCAACCA |
| Tpm1-AS-R: | TGCGGGAAACCGCTGAAGCTGA |
| Tpm1-M-R: | AGGCCACAGATGCTGAAGCTGA |
| RIP-qPCR primers | |
| Tpm1-F | ACATTGCCACACTTCACAGAG |
| Tpm1-R | TGTCTCCAACATCAGTCTTCCT |
| Ccnb1-F | CTGCCGAAGAATATCTGTGTCA |
| Ccnb1-R | CTCCAGTTGTCTGAGGTAAGC |
| Ccnd1-F | AGAAGTGCGAAGAGGAGGTC |
| Ccnd1-R | GCGGATAGAGTTGTCAGTGTAG |
| Internal control | |
| GAPDH-F | TGCCACTCAGAAGACTGTGG |
| GAPDH-R | TTCAGCTCTGGGATGACCTT |
